# Interference of Small Sequence Variants with MLPA in *CLCN1*: Implications for Congenital Myotonia Diagnosis

**DOI:** 10.3390/genes17091073

**Published:** 2026-09-05

**Authors:** Maria Busacca, Eleonora Canioni, Raffaella Brugnoni

**Affiliations:** Neurology IV Unit, Neuroimmunology and Neuromuscular Diseases, Fondazione IRCCS Istituto Neurologico Carlo Besta, 20133 Milan, Italy; maria.busacca@istituto-besta.it (M.B.); eleonora.canioni@istituto-besta.it (E.C.)

**Keywords:** congenital myotonia, Becker’s disease, *CLCN1*, MLPA, Copy Number Variations, exonic deletion, false positive

## Abstract

Background/Objectives: Congenital myotonia (CM) is an inherited neuromuscular disorder caused by mutations in the Chloride Voltage-Gated Channel 1 (*CLCN1*) gene, encoding the Chloride Channel 1 (CIC-1) in skeletal muscle. These mutations can be inherited in either an autosomal dominant (Thomsen’s disease) or recessive pattern (Becker’s disease). Within the diagnostic workflow for this pathology, gene sequencing represents one of the main analytical approaches, while Multiplex Ligation-dependent Probe Amplification (MLPA) is used as a complementary method, particularly in patients with autosomal recessive inheritance, to detect deletions or duplications involving one or more exons of the *CLCN1*. This study aims to highlight a specific limitation of MLPA in *CLCN1* analysis. Methods: Patients carrying pathogenic *CLCN1* variants, previously identified by sequencing, subsequently underwent MLPA analysis to investigate the presence of a second pathogenic allele. Results: In the analysed patients, MLPA identified apparent exon deletions involving the same exons in which the previously identified variants were present. However, no actual variation in copy number was observed at the exon level. This discrepancy is linked to the efficiency of probe hybridization and ligation to target sequences. Small sequence variations can interfere with probe binding, preventing their correct ligation and resulting in a signal pattern consistent with apparent exon loss, thereby generating false positives. Conclusions: Our findings highlight the importance of integrating sequencing and MLPA data in *CLCN1* analysis. Awareness of variant-induced MLPA artefacts is essential to avoid misinterpretation of apparent exon deletions and to ensure an accurate molecular diagnosis of CM.

## 1. Introduction

Congenital myotonia (CM) is a hereditary muscle disease mainly characterised by muscle hyperexcitability, muscle stiffness, and hypertrophy [1]. Congenital myotonia is the most common form of non-dystrophic myotonia and is caused by mutations in the Chloride Voltage-Gated Channel 1 (*CLCN1*) gene [2], located on chromosome 7q35 [3], which encodes the voltage-gated Chloride Channel 1 (CIC-1) in skeletal muscle [4], an essential regulator for maintaining normal muscle membrane excitability [5]. Mutations in *CLCN1* result in reduced chloride conductance [6], altering the repolarisation mechanisms of the muscle fibre and causing a state of hyperexcitability, leading to prolonged and sustained muscle contraction (myotonia) [7].

Depending on the pattern of inheritance, CM can be classified into an autosomal dominant form (Thomsen’s disease, DCM) (OMIM: 160800) and an autosomal recessive form (Becker’s disease, RCM) (OMIM: 255700), which differ in their clinical phenotypes [3], such as age of onset and degree of myotonia [7]. RCM is more common and clinically more severe than DCM [3]. It has also been observed that some mutations may exhibit incomplete penetrance or variable expressivity, contributing to the clinical heterogeneity of the condition [5].

Epidemiologically, the prevalence of DCM is approximately 1 in 23,000 individuals, while that for RCM is approximately 1 in 50,000 [7]. Over 300 pathogenic variants have been described to date [8,9], distributed across the 23 exons and the intronic regions of *CLCN1* [3]. Most of the identified mutations are point variants, particularly missense and nonsense mutations, while small insertions/deletions and larger genomic rearrangements are less common [2]. Mutations associated with RCM are often present in compound heterozygosity [10].

From a diagnostic perspective, it is also important to take into account the differential diagnosis with other skeletal muscle channelopathies, in particular sodium channel myotonia (SCM; OMIM #170500 and #613345) [7], caused by pathogenic variants in the Sodium Voltage-Gated Channel Alpha Subunit 4 (*SCN4A*) gene, which encodes the α subunit of the voltage-gated sodium channel in skeletal muscle. *SCN4A* mutations generally result in a gain-of-function of the channel and can give rise to clinical manifestations that overlap with those of CM [2]. Furthermore, it has been estimated that up to 20% of patients suspected of having CM actually carry pathogenic variants in the *SCN4A* [11].

Once pathogenic variants in the *SCN4A* have been excluded by sequencing, molecular diagnosis of CM involves the sequencing of *CLCN1* [7], followed by analysis to detect deletions or duplications affecting one or more exons within the gene [2]. This complementary analysis is usually carried out using Multiplex Ligation-dependent Probe Amplification (MLPA), a molecular technique based on multiplex PCR amplification [12]. Its widespread use is due to its ability to provide a rapid, sensitive and affordable analysis of Copy Number Variations (CNVs), enabling the assessment of numerous genomic regions in a single reaction [13].

MLPA is an important diagnostic technique for the characterisation of RCM cases, enabling the detection of deletions and duplications in the *CLCN1* [2]. The method allows the quantification of all 23 exons of the gene, making it the preferred tool for identifying CNVs that cannot be detected by conventional sequencing [14]. Despite its high reliability in identifying exonic deletions and duplications, MLPA has some intrinsic limitations [15]. This technique is designed to detect CNVs and does not determine the precise extent of rearrangements or the characterisation of nucleotide alterations [16]. Furthermore, as the correct functioning of the method depends on the hybridisation and subsequent ligation of the probes [17], the presence of point variants or small insertions/deletions in the binding regions can compromise the efficiency of the reaction [15]. Consequently, a reduction in the signal generated by the probe may be attributed to the presence of an apparent exonic deletion [18], leading to confusing results [13].

Although the interference of sequence variants with MLPA probe hybridization is a recognised phenomenon [13,15], its diagnostic implications in *CLCN1*-related CM have received limited attention. This issue is particularly relevant in patients with suspected RCM in whom sequencing identifies only a single pathogenic variant, prompting the use of MLPA to investigate the presence of a second disease-causing allele.

The aim of this study is to assess the effects of small *CLCN1* mutations on MLPA results and to illustrate how such alterations, which affect the probe-binding sites, can result in apparent deletions of entire exons, leading to false-positive interpretations and highlighting the need for a combined analysis of sequencing and MLPA data in the diagnostic workflow for RCM.

## 2. Materials and Methods

### 2.1. Sample Collection

The study was conducted according to the principles outlined in the Declaration of Helsinki. All patients signed an informed consent form for genetic analysis. Genomic DNA was extracted from peripheral blood on Freedom Evo-2 100 Base (Tecan, Männedorf, Switzerland) using a NucleoSpin Blood Kit following the manufacturer’s instructions (Macherey-Nagel, Düren, Germany).

### 2.2. Sequencing Analysis

Sequencing of the *CLCN1 gene* (GenBank accession number: NM_000083.3) in all four patients analysed in this study was previously carried out as part of the routine diagnostic workflow using Next-Generation Sequencing (NGS), following the protocol previously described [14]. All patients with a clinical suspicion of CM referred to our diagnostic laboratory have been routinely analysed using a targeted NGS panel including the *CLCN1* and *SCN4A* genes [14]. MLPA analysis was subsequently performed only in patients carrying a single *CLCN1* variant, either novel or previously reported as recessive, in order to investigate the presence of a second pathogenic variant. Among these cases, only one patient was found to harbour a true multi-exonic *CLCN1* deletion, whereas 11 patients showed an apparent deletion of exons 5, 11, and 13 by MLPA. Three representative cases were selected from this group for inclusion in the present study.

### 2.3. MLPA Analysis

The MLPA analysis was performed using the SALSA MLPA Probemix P350 *CLCN1*-*KCNJ2* kit (MRC Holland, Amsterdam, The Netherlands), according to the manufacturer’s instructions and the original protocol described by Schouten et al. [12], containing probes specific to all 23 exons of the *CLCN1.* Genomic DNA was first denatured and then incubated with a mixture of specific oligonucleotide probes designed to hybridise to adjacent target sequences. Following the hybridisation step, the adjacent probe segments were joined via a ligation reaction. Only the correctly ligated probes were subsequently amplified by multiplex PCR using a universal pair of primers, one of which was labelled with a fluorochrome. The amplification products were separated by capillary electrophoresis on an ABI PRISM 3130xl Genetic Analyser (Applied Biosystems, Foster City, CA, USA) and quantified by analysing the intensity of the fluorescent peaks using Coffalyser.Net software v.250317.1029 (MRC Holland), which compares each sample with three reference samples (healthy individuals) processed under the same experimental conditions. Values between 0.7 and 1.3 were considered consistent with a normal copy number, while values close to 0.5 and 1.5 were interpreted as indicative of heterozygous deletion and duplication, respectively. For the purpose of this study, representative cases showing discordant results between sequencing and MLPA analyses were selected, together with one case carrying a confirmed multi-exon deletion.

## 3. Results

MLPA analysis performed in patients with suspected RCM revealed monoallelic deletions in the *CLCN1* in four cases. Three patients carried an apparent deletion involving a single exon (5, 11 and 13, respectively), whereas one patient harboured a deletion spanning exons 21, 22, and 23 (Table 1).

**Figure 1:**
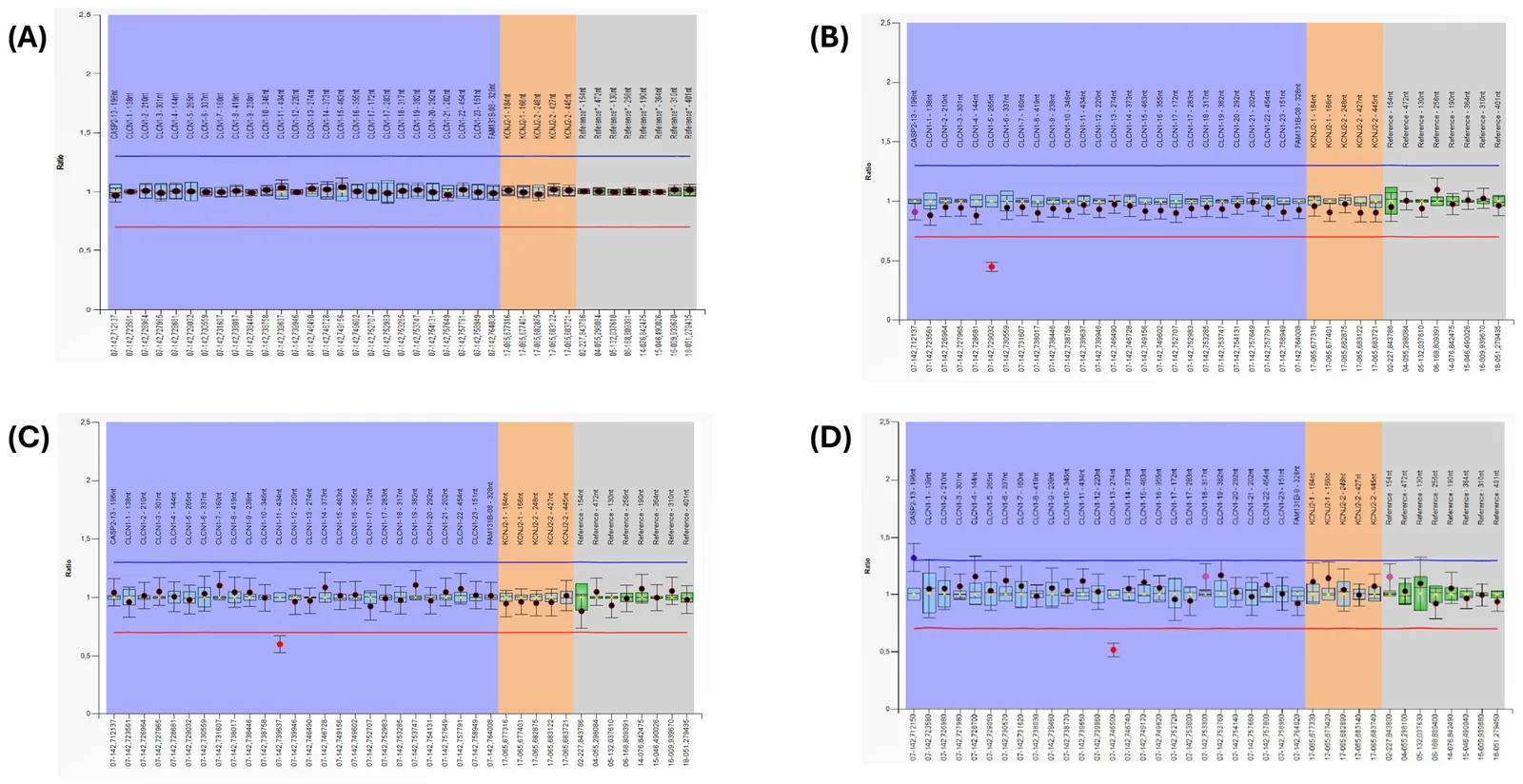
Ratio charts of the results of congenital myotonia (CM) samples analysed with the SALSA MLPA Probemix P350 *CLCN1*-*KCNJ2*. Red dots display the probe dosage ratios and the error bars show the 95% confidence ranges. The grey box plot in the background shows the 95% confidence range of the reference samples used for normalization. Probes targeting Chloride Voltage-Gated Channel 1 (*CLCN1*) and Rectifying Channel Subfamily J Member 2 (*KCNJ2*) are indicated by the blue/purple and orange shaded background areas, respectively. Probe locations are displayed on the X-axis and dosage ratios on the Y-axis. The red and blue lines at ratios 0.7 and 1.3 indicate the arbitrary borders for copy number loss and gain, respectively. The displayed samples show a reduced Multiplex Ligation-dependent Probe Amplification (MLPA signal for *CLCN1* exon 5 (**B**), 11 (**C**) and 13 (**D**), and a reference control sample showing normal dosage ratios across all *CLCN1* and *KCNJ2* probes (**A**). The experiment was performed according to the manufacturer’s MLPA standard protocol and data were analysed with the Coffalyser.Net provided by MRC Holland (Amsterdam, The Netherlands). Additional coloured symbols and boxes are automatically generated by Coffalyser.Net for data visualisation and quality control purposes and were not used for result interpretation.

**Figure 2:**
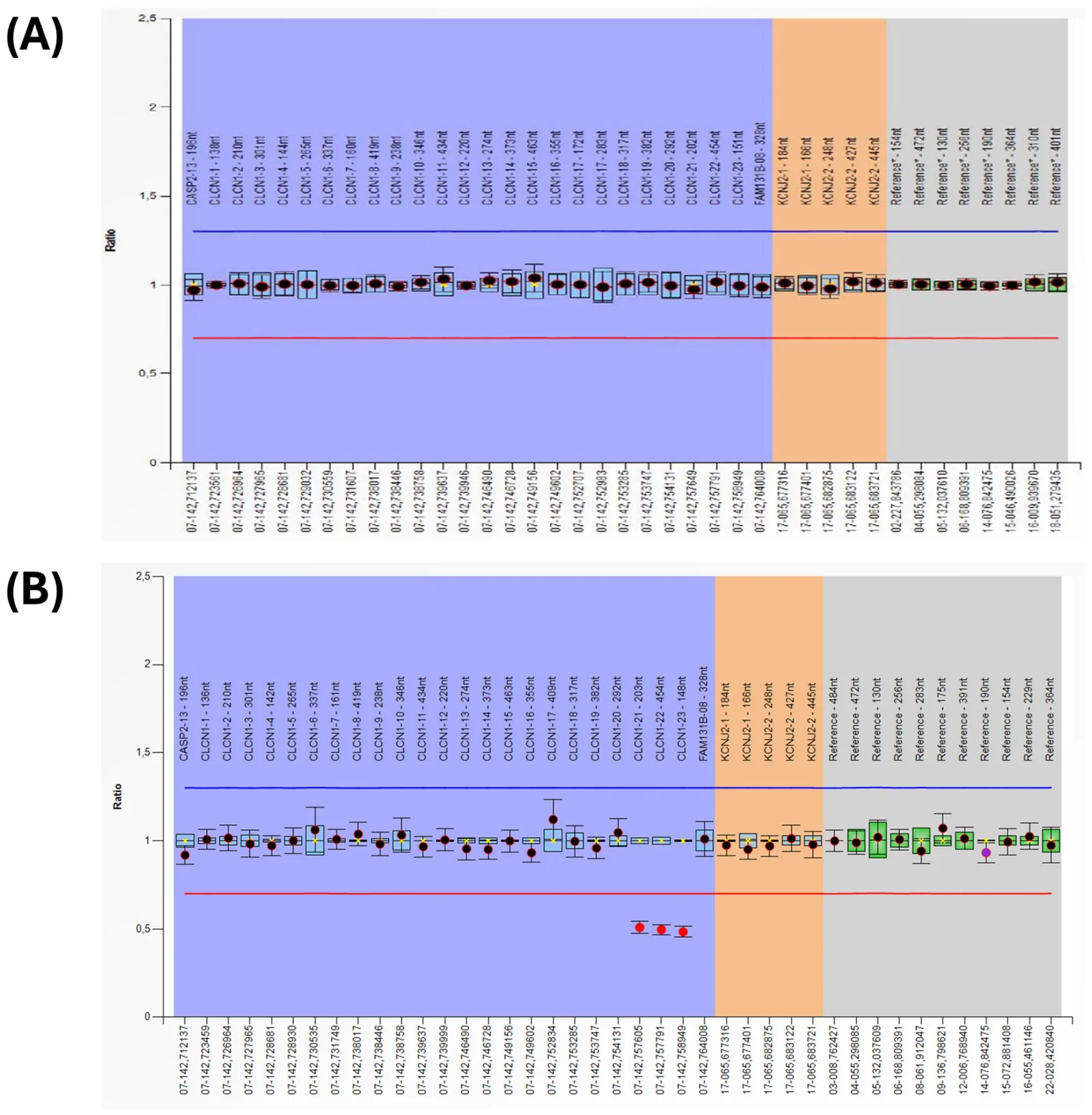
Ratio charts of the results of congenital myotonia (CM) samples analysed with the SALSA MLPA Probemix P350 *CLCN1*-*KCNJ2*. Red dots display the probe dosage ratios and the error bars show the 95% confidence ranges. The grey box plot in the background shows the 95% confidence range of the reference samples used for normalization. Probes targeting Chloride Voltage-Gated Channel 1 (*CLCN1*) and Rectifying Channel Subfamily J Member 2 (*KCNJ2*) are indicated by the blue/purple and orange shaded background areas, respectively. Probe locations are displayed on the X-axis and dosage ratios on the Y-axis. The red and blue lines at ratios 0.7 and 1.3 indicate the arbitrary borders for copy number loss and gain, respectively. The displayed samples show the deletion of *CLCN1* exon 21-23 (**B**) and a reference control sample showing normal dosage ratios across all *CLCN1* and *KCNJ2* probes (**A**). The experiment was performed according to the manufacturer’s Multiplex Ligation-dependent Probe Amplification (MLPA) standard protocol and data were analysed with the Coffalyser.Net provided by MRC Holland (Amsterdam, The Netherlands). Additional coloured symbols and boxes are automatically generated by Coffalyser.Net for data visualisation and quality control purposes and were not used for result interpretation.

Figure 1A and Figure 2A show the MLPA profile of a reference sample, characterised by a normal signal pattern for all the exons of *CLCN1*, with values included within the expected range (0.7–1.3), consistent with a normal copy number for the sequences detected and indicative of the correct performance of the CM patient’s analysis. In the analysed pathological samples, the MLPA results showed a signal reduction for specific exons, suggesting the presence of heterozygous deletions (ratio ~0.5). In accordance with the technique’s interpretative criteria, this reduction is consistent with the apparent deletion of an entire exon in the heterozygous form [18].

The sequencing analysis of Patient 1 revealed the known heterozygous missense variant c.568_569GG > TC (p.G190S) in exon 5 of the *CLCN1* (NM_000083.3), described in the literature in association with both DCM and RCM [14,19,20,21,22,23,24,25]. However, the MLPA profile indicated an apparent heterozygous deletion of the entire exon 5 in the same gene (Figure 1B) (Table 1).

In Patient 2, carrying the known heterozygous deletion c.1182_1186delGGAAT (p.G395Cfs*32) in exon 11 of the *CLCN1* (NM_000083.3) and previously identified in RCM patients [14,25,26,27], MLPA revealed an apparent heterozygous deletion pattern in the same exon 11 (Figure 1C) (Table 1).

Patient 3 carried the known intronic deletion c.1471 + 4delTCAAGAC, located 4 nucleotides downstream of exon 13 of the *CLCN1* (NM_000083.3) and previously identified in a patient with RCM [14], and showed an apparent loss in a heterozygous form of exon 13 according to the MLPA analysis (Figure 1D).

In contrast, in patient 4, carrying the known heterozygous variant c.1444G > A (p.G482R) in exon 13 of *CLCN1* (NM_000083.3) [28,29], MLPA analysis revealed a heterozygous deletion of the last three exons of the *CLCN1* (exons 21, 22 and 23) (Figure 2B) (Table 1).

For Patients 1–3, the apparent exon losses were consistent with MLPA artefacts because the sequence variants identified by NGS were located within or close to the MLPA probe target regions and no additional evidence supporting a true exon deletion was observed.

In particular, Patient 1 carried the c.568_569GG > TC (p.G190S) variant in exon 5 of *CLCN1*, a recurrent variant frequently observed in our Italian cohort and previously reported by our group [14]. In addition to the index case, we identified six other unrelated patients with RCM, carrying the same c.568_569GG > TC (p.G190S) variant, who consistently showed the same apparent exon 5 deletion by MLPA.

Similarly, for Patient 2, the c.1182_1186delGGAAT variant and the corresponding apparent exon 11 deletion were also detected in the patient’s father and in an additional unrelated patient [14].

For Patient 3, who carried an apparent exon 13 deletion and the c.1471 + 4delTCAAGAC variant, the latter was previously identified as a novel variant in our Italian cohort study [14]. However, no additional family members were available for segregation analysis.

In contrast, the multi-exon deletion involving exons 21–23 identified in Patient 4 was confirmed upon repeat MLPA analysis. As previously reported in our study [28], the same deletion affecting the last three exons of *CLCN1* was also detected by MLPA in one of the patient’s parents, supporting its segregation within the family and confirming that this represents a true genomic deletion rather than an artefactual MLPA finding.

## 4. Discussion

Congenital myotonia (CM) is an inherited skeletal muscle channelopathy characterised by high genetic heterogeneity [5], with over 300 pathogenic variants described in the Chloride Voltage-Gated Channel 1 (*CLCN1*) gene [8,9].

In the diagnostic workflow for recessive CM (RCM) [2], Multiplex Ligation-dependent Probe Amplification (MLPA) is commonly used as a complementary method to Next Generation Sequencing (NGS) or Sanger sequencing [13,30], particularly when only a single pathogenic variant has been identified and it is necessary to identify Copy Number Variations (CNVs) in the other allele not detected by conventional sequencing [14]. Although depth-of-coverage analysis from NGS data is increasingly used to identify exon-level CNVs, its performance depends on several factors, including sequencing platform characteristics, panel design, coverage uniformity, bioinformatic pipelines, and laboratory-specific validation procedures [31]. Therefore, MLPA remains a widely accepted and reliable method for CNV confirmation in many diagnostic laboratories [13]. The variants examined in this study have previously been described in the literature as pathogenic variants associated with autosomal recessive forms of CM [14,19,20,21,22,23,24,25,26,27,28,29], thereby confirming the usefulness of MLPA as a second-level test in the search for a further pathogenic allele.

However, the results obtained in this work have highlighted an important aspect regarding the use of MLPA for the analysis of *CLCN1*. In some cases, MLPA analysis showed a signal reduction consistent with an apparent heterozygous deletion of the same exon containing the variant identified by sequencing. This phenomenon was observed in Patients 1–3 in exons 5, 11 and 13, where the apparent deletion detected by MLPA did not correspond to the actual loss of genetic material, but was associated with the presence of small alterations identified by sequencing.

This discrepancy depends on the correct pairing and subsequent ligation of two probes of MLPA to specific regions of the target DNA [17]. Sequence variants located within or close to probe-binding regions may reduce the MLPA signal, generating a result consistent with a copy number variation and, therefore, generating a false positive [15].

This mechanism is directly supported by the c.1182_1186delGGAAT variant in exon 11, which falls exactly within the target region of the corresponding MLPA probe. In particular, the manufacturer specifically states that this variant is able to affect the probe’s signal generation and recommends sequencing the target region in cases of apparent exon deletion. According to the manufacturer, sequence variants located within a probe’s target sequence can reduce the MLPA signal by preventing ligation or destabilising probe hybridisation, even when located more than 20 nt from the ligation site. Therefore, this mechanism could explain the apparent exon losses observed in patients carrying variants in exons 5 and 13, although the respective variants were not located within MLPA probes’ target regions.

It is important to stress that this phenomenon does not represent an error in the method, but rather a consequence of its intrinsic characteristics.

Particular caution is required when an apparently homozygous variant is identified within an exon showing reduced MLPA signal, as this finding may reflect hemizygosity due to a deletion on the second allele rather than true homozygosity. In such cases, segregation studies and/or independent CNV confirmation methods are essential to establish the correct molecular diagnosis.

Our data highlights the need for careful interpretation of isolated single-exon deletions detected by MLPA in *CLCN1*. This is especially important in the diagnostic workup of patients with suspected RCM who carry only one pathogenic variant identified by NGS or Sanger sequencing, frequently inherited from an asymptomatic parent. In such cases, apparent exon deletions may actually reflect sequence variants located within MLPA probe-binding regions, underscoring the importance of integrating MLPA results with sequencing data before establishing a molecular diagnosis. Therefore, evaluating MLPA results in relation to the information obtained through sequencing is significantly relevant in the *CLCN1*, where small mutations represent the most frequently observed category of variants [2,9].

## 5. Conclusions

In conclusion, MLPA findings suggestive of single-exon deletions should always be interpreted in conjunction with sequencing results. Repetition of the MLPA assay is recommended as a first measure to confirm the consistency of the result. In addition, whenever family samples are available, segregation analysis may provide valuable supportive evidence to clarify the clinical significance of the finding. If uncertainty persists, confirmation by independent methods should be considered before concluding that a true copy number variation is present. Among the complementary analyses available for CNV confirmation, quantitative PCR (qPCR) is one of the most widely used methods in diagnostic laboratories. By targeting genomic regions independently of MLPA probe-binding sites, qPCR can help distinguish true exon deletions from apparent copy number losses resulting from sequence variants that interfere with MLPA probe hybridization or ligation [32].

In unresolved cases, Whole Exome Sequencing (WES) and Whole Genome Sequencing (WGS) may help identify pathogenic variants outside the regions interrogated by MLPA or detect copy number changes through dedicated bioinformatic analyses [33], while RNA sequencing (RNA-seq) may reveal aberrant splicing events and transcript-level defects [34]. Together, these approaches can overcome some of the intrinsic limitations of MLPA and contribute to a more comprehensive molecular characterisation of *CLCN1*-related disease.

Awareness of this phenomenon is essential to avoid misinterpretation of MLPA findings and to ensure an accurate molecular diagnosis of *CLCN1*-related congenital myotonia.

## Figures and Tables

**Table 1:** Summary of Chloride Voltage-Gated Channel 1 (*CLCN1*) variants identified by sequencing analysis and corresponding Copy Number Variation (CNV) detected by Multiplex Ligation-dependent Probe Amplification (MLPA).

Patient	Sequence Analysis *	*CLCN1* Region	MLPA Analysis	*CLCN1* Region
1	c.568_569GG > TC	Exon 5	“Apparent” deletion of 1 exon	Exon 5
2	c.1182_1186delGGAAT	Exon 11	“Apparent” deletion of 1 exon	Exon 11
3	c.1471 + 4delTCAAGAC	Intron 13	“Apparent” deletion of 1 exon	Exon 13
4	c.1444G > A	Exon 13	Deletion of 3 exons	Exons 21–23

* Sequencing revealed heterozygous pathogenic variants in all four patients, while MLPA analysis identified apparent heterozygous exon deletions affecting either the same exon harbouring the sequence variants (Patients 1–3) or heterozygous deletion encompassing the last three exons of *CLCN1* (exons 21–23) (Patient 4).

## Data Availability

The data presented in this study are openly available in repository [10.5281/zenodo.10880296], upon reasonable request.

## Abbreviations

The following abbreviations are used in this manuscript:

CIC-1	Chloride Channel 1
*CLCN1*	Chloride Voltage-Gated Channel 1
CM	Congenital Myotonia
CNVs	Copy Number Variations
DCM	Dominant Congenital Myotonia
*KCNJ2*	Potassium Inwardly Rectifying Channel Subfamily J Member 2
MLPA	Multiplex Ligation-dependent Probe Amplification
NGS	Next Generation Sequencing
qPCR	quantitative PCR
RCM	Recessive Congenital Myotonia
RNA-seq	RNA sequencing
*SCN4A*	Sodium Voltage-Gated Channel Alpha Subunit 4
WES	Whole Exome Sequencing
WGS	Whole Genome Sequencing
